# Morphology and Dynamics in Hydrated Graphene Oxide/Branched Poly(ethyleneimine) Nanocomposites: An In Silico Investigation

**DOI:** 10.3390/nano13121865

**Published:** 2023-06-15

**Authors:** Anastassia Rissanou, Apostolos Konstantinou, Kostas Karatasos

**Affiliations:** 1 Theoretical & Physical Chemistry Institute, National Hellenic Research Foundation, 48 Vassileos Constantinou Avenue, 11635 Athens, Greece; trissanou@eie.gr; 2 Chemical Engineering Department, Aristotle University of Thessaloniki, 54124 Thessaloniki, Greece

**Keywords:** molecular dynamics simulations, graphene oxide, poly(ethyleneimine), nanocomposites

## Abstract

Graphene oxide (GO)—branched poly(ethyleneimine) (BPEI) hydrated mixtures were studied by means of fully atomistic molecular dynamics simulations to assess the effects of the size of polymers and the composition on the morphology of the complexes, the energetics of the systems and the dynamics of water and ions within composites. The presence of cationic polymers of both generations hindered the formation of stacked GO conformations, leading to a disordered porous structure. The smaller polymer was found to be more efficient at separating the GO flakes due to its more efficient packing. The variation in the relative content of the polymeric and the GO moieties provided indications for the existence of an optimal composition in which interaction between the two components was more favorable, implying more stable structures. The large number of hydrogen-bonding donors afforded by the branched molecules resulted in a preferential association with water and hindered its access to the surface of the GO flakes, particularly in polymer-rich systems. The mapping of water translational dynamics revealed the existence of populations with distinctly different mobilities, depending upon the state of their association. The average rate of water transport was found to depend sensitively on the mobility of the freely to move molecules, which was varied strongly with composition. The rate of ionic transport was found to be very limited below a threshold in terms of polymer content. Both, water diffusivity and ionic transport were enhanced in the systems with the larger branched polymers, particularly with a lower polymer content, due to the higher availability of free volume for the respective moieties. The detail afforded in the present work provides a new insight for the fabrication of BPEI/GO composites with a controlled microstructure, enhanced stability and adjustable water transport and ionic mobility.

## 1. Introduction

One of the major tasks related to the fabrication of three-dimensional, porous materials is to enhance their structural stability and to control the interactions that drive the microscopic organization of their constituents [1]. A versatile strategy to achieve this goal is the combination of inorganic (i.e., “hard”) fillers with polymeric (i.e., “soft”) building blocks to produce hybrid materials with desired structural features and functionalities [2]. In the last two decades, graphene and its derivatives have emerged as appropriate “hard” fillers that can be dispersed in polymeric matrices, yielding nanocomposite materials with enhanced thermal and mechanical stabilities [3,4,5,6,7,8]. Although several synthetic protocols have been developed in order to produce graphene-based polymer hybrids with a well-defined nanostructure [9,10,11], the control of the mechanisms related to the assembly of graphene-based nanosheets in order to suppress aggregation, while preserving the desired functionalities, still remains challenging [12,13,14,15]. Several of the proposed synthetic routes involve the dispersion of graphene and its derivatives in liquid media, aiming at stabilized the mixtures, which may enable viable processing conditions and lead to the production of composite materials with properties that are appropriate for final applications [16,17,18].

In particular, when aqueous media are used for this purpose, apart from the role of the chemical composition of graphene-based fillers in overcoming hydrophobic interactions, i.e., the degree of oxidation [19,20,21] and functionalization [22,23,24], the presence of polyelectrolytes is known to facilitate the formation of dispersions with controlled 3D structures via electrostatic stabilization [25,26,27]. A category of polymer electrolytes that have commonly been utilized as solubilization and structure-enhancement agents in the aqueous dispersion of oxidized forms of graphene are branched molecules with a dendritic topology [28,29,30,31,32,33,34]. These molecules contain many functional groups within a small volume, allowing there to be multiple interacting sites with graphene-based fillers, even when small-molecular-weight polymers are used [35,36,37]. At neutral pH conditions where the graphene oxide (GO) flakes are negatively charged [38,39], complexation with cationic dendritic polyelectrolytes towards the formation of nanoporous 3D structures is primarily driven by favorable electrostatic interactions [23,28,40,41]. Another mechanism that contributes significantly to the polymer/GO affinity, and thus facilitates the formation of structurally and energetically stable composite materials, is hydrogen bonding. The large specific area of the graphene-based sheets in combination with the presence of oxidized groups (such as carboxyls, hydroxyls and epoxides), enhance the physical adsorption of branched electrolytes, which can dispose of hydrogen-bonding donors or acceptors [36,42,43,44]. The presence of multiple non-bonded interactions between cationic branched polymers and GO can be exploited for the fabrication of nano-hybrids with self-healing properties [43,44]. Moreover, the reversible nature of these non-covalent interactions in the formed complexes facilitate the regeneration of these materials in a cost-effective manner [36,42,45,46], thus overcoming the recycling difficulties often present in the retrieval of GO-based materials from aqueous media [36].

In several of the applications in which complexes between GO and branched cationic polyelectrolytes have been used, the control of spacing between the GO flakes, and thus of the microporosity of the formed structure, is of great importance [47,48,49]. This can be manipulated by embedding branched molecules with varying sizes or topologies between the GO flakes [50]. Although branched molecules of relatively high molecular weight (i.e., larger than 50,000 Daltons) have been utilized for this purpose [36], the use of large molecular weights is not appropriate if a higher degree of GO loading with polymer is intended [36], or when these materials are destined for biomedical applications [51]. For nanometer-size separations, low-molecular-weight dendritic molecules (i.e., of the order of 1000 Daltons or lower) can be employed [28,50]. There are experimental indications that a smaller size of the branched polymers allows easier diffusion [12] and better interdigitation between the GO flakes [35], resulting in more stable structures. The fine tuning of branched polymer/GO microstructure can then be performed by changing parameters, such as the relative proportions of the components [52,53], the size and the aspect ratio of the flakes [54] or the dimensions of the branched polymers in the composites [28].

In this work, we performed fully atomistic molecular dynamics (MD) simulations to explore the effects of the composition of GO/poly(ethylenimine) (BPEI) hydrated composites on the resulting microstructure and its impact on the dynamic behavior of water molecules and counterions. GO membranes and aqueous dispersions comprising nanosized flakes have previously been fabricated [55,56], while composites produced by mixing BPEI polymers and GO have demonstrated their potential as materials with high-end uses [12,46]. To check the possible effects of the size of the branched polymers on inter-flake separation and the packing behavior of polymers near the GO surface, we studied systems comprising low-molecular-weight branched poly(ethyleneimine) of two different generations. In addition, we examined, in detail, the role of hydrogen bonding in the associative behavior between components of the mixture.

## 2. Materials and Methods

### 2.1. Simulated Models

**Figure 1:**
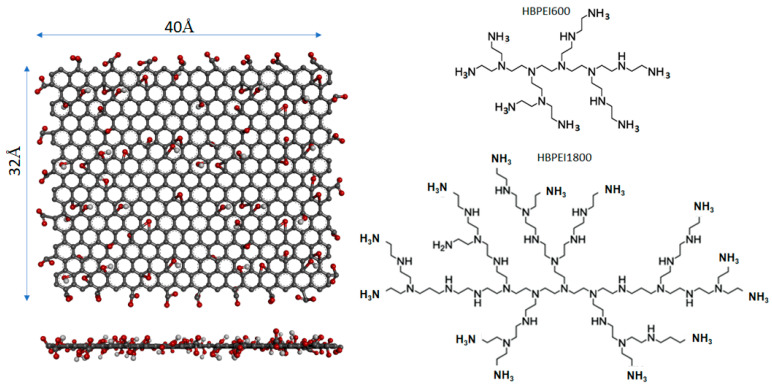
Illustration of the structure of the GO sheet (**left**) and the branched BPEI molecules (**right**) used in the simulations. The GO sheet is shown in frontal and lateral projections. Carbon atoms are shown in gray color, oxygen atoms are shown in red and hydrogen atoms are shown in white.

We have simulated composite systems based on the non-covalent association between GO nanoflakes and branched poly(ethyleneimine) molecules of two different generations (i.e., 2nd and 3rd) in the presence of water and counterions. The oxidation scheme of GO flakes followed the Lerf–Klinowski model [57]. According to this model, hydroxyl and epoxy groups are randomly placed on both sides of the basal graphene plane, while carboxyl groups are preferably located on the edges of the flake. In the construction of a GO flake, one carboxyl group was placed every 20 peripheral carbons [58], the ratio of the hydroxyl to epoxy groups was kept to 3:2, and the overall carbon to oxygen ratio was approximately 5:1. To simulate a neutral pH environment, the peripheral carboxyl groups of the flakes were ionized, while the hydroxyls were left protonated [39]. This procedure resulted in a net charge for each GO flake of −26|e|. To mimic the same pH conditions for the branched polymers, the primary amines of the BPEI molecules were also protonated [59]. After protonation, the net charge for a 2nd generation polymer was +7|e|, and that of the 3rd generation one was +14|e|. Figure 1 shows the structure of the GO flakes and the BPEI polymers used in the present work.

We constructed 3 systems at varying compositions based on each of the two BPEI generations. This allowed the investigation of composites during which the polymer or the GO was the majority phase, as well as the case in which the two components were mixed in similar proportions [53]. An appropriate number of Cl^−^ counterions were included in each model to preserve the overall electrical neutrality. The water content varied at 5%wt–10%wt. The examined systems mimicked GO nanocomposites with enhanced mechanical properties [60]. Table 1 provides details on the composition of all systems. The somewhat different amounts of water present in the analogous systems, i.e., S1/T1, S2/T2 and S3/T3 were necessary in order to achieve similar polymer weight fractions at an almost constant water content.

### 2.2. Simulation Method and Protocol

Initial configurations for all systems were constructed via randomly inserting the different constituents of each model using the corresponding tool in GROMACS software [61]. Initial configurations of the systems are shown in Figure S1 in the Supplementary Material. Energetic parameters describing the bonded and non-bonded interactions for GO and BPEI molecules were adopted from the OPLSAA forcefield [62] while the TIP3P explicit solvent model was used for water [63]. The OPLSAA forcefield in conjunction with the TIP3P model for water has previously been utilized for the description of oxidized forms of graphene [64,65], branched PEI polymers [66] and Cl^−^ ions [64] in aqueous media. All simulations were performed using the GROMACS package [61], with periodic boundary conditions applied in all dimensions. A cutoff of 1.0 nm was used for van der Waals and real-space electrostatic interactions, while the Particle Mesh Ewald (PME) method [67] was employed for the calculation of long-range electrostatic forces. Long-range corrections were also considered for van der Waals interactions.

**Figure 2:**
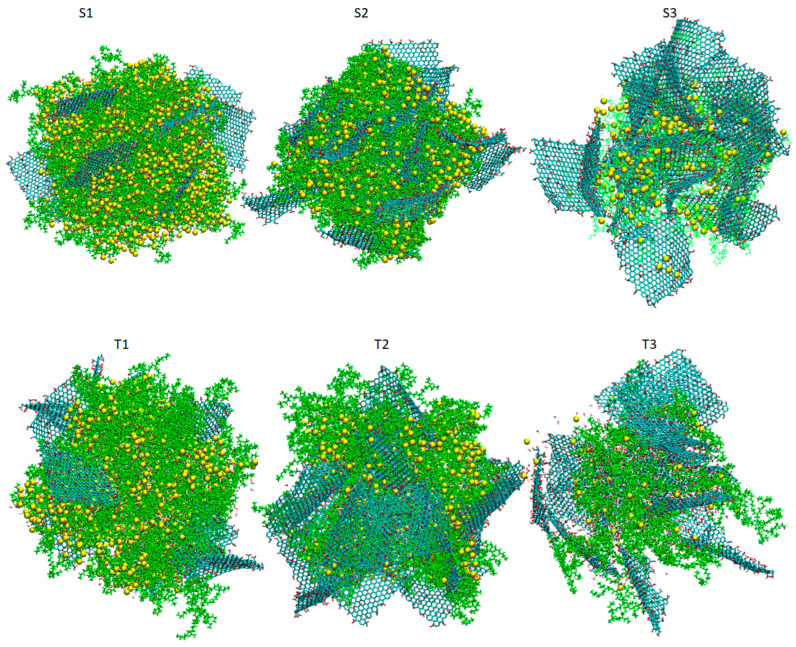
Snapshots of the simulated systems after equilibration. Periodic boundary conditions have been taken into account in the depiction of the models. Carbon atoms in the GO flakes appear in dark cyan, oxygen atoms are shown in red, hydrogens are shown in white, BPEI polymers are shown in green and Cl^−^ counterions appear as yellow beads. Water atoms appear as dots.

Simulation trajectories of 150 ns length were generated according to the following protocol, which comprised three steps: After the construction of the initial configurations, energy minimization was performed. Next, to facilitate the mixing of the two components an annealing procedure was applied starting from 550 K and reaching room temperature (i.e., 300 K), with a rate of 2.5 K/ns. Production runs in the isobaric–isothermal (NPT) ensemble followed for 40 ns, and we used the Nose–Hoover thermostat [68] to keep the temperature constant at 300 K and the Parrinello–Rahman barostat [69] to maintain the pressure at 1 atm. The time step for the integration of equations of motion was 1 fs, whereas bonds involving hydrogens were constrained during the simulation using the LINCS algorithm [70]. The time evolution of characteristic structural measures of the models subjected to the adopted protocol is presented in Figure S2. Figure 2 depicts the configurations of the systems after equilibration.

## 3. Results and Discussion

### 3.1. Spatial Arrangement of the Components

**Figure 3:**
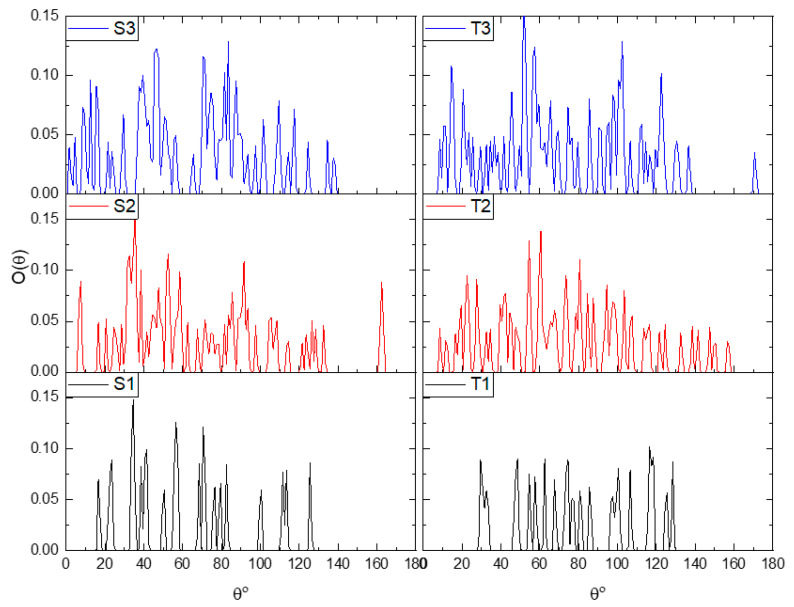
Angle distributions referring to the relative orientation of the GO flakes (see text) in the examined systems.

A visual inspection of the snapshots in Figure 2 shows that GO does not adopt configurations with a parallel (or almost parallel) arrangement of flakes. The average properties are based on the analysis of the equilibrated part of the trajectory. To check the average behavior over the equilibrated trajectory for each system, we calculated the distribution of the angles, O(θ), where θ denotes the angle between the eigenvectors normal to the GO plane for a pair of flakes, as these were determined via the diagonalization of the moment of inertia tensor of each flake. The so-calculated angle distributions are plotted in Figure 3. Evidently, there are no dominant peaks close to θ = 0° or θ = 180° for any of the examined systems. Therefore, on average, there is no tendency of the GO flakes to assume stacked configurations. The distributions spread out within a wide range of angles, indicating the practically random relative orientation of flakes. In the absence of the polymeric component, GO in aqueous media is expected to show a tendency for the formation of clusters within which the flakes can be oriented in parallel [19]. Therefore, this observation should be directly associated with the presence of BPEI molecules [53]. The development of a network formed by orientationally disordered GO flakes due to their interaction with branched PEI polymers has already been experimentally observed [36] and found to lead to the formation of stable three-dimensional structures [46,53].

**Figure 4:**
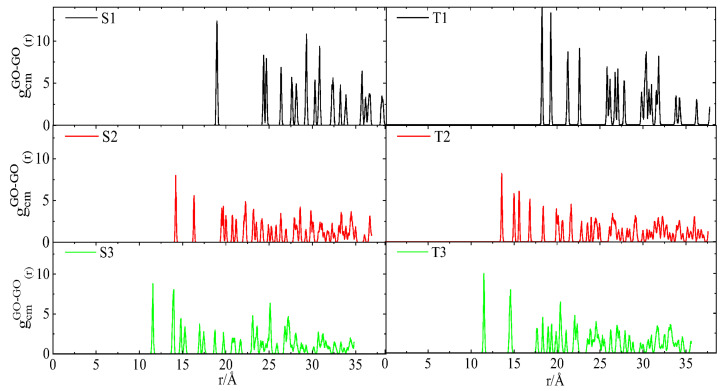
Radial distribution functions arising from the centers of mass of the GO flakes.

In the absence of the polymeric moiety and at a high water content, this distribution is expected to be smooth curve [19]. The discretized form of distributions in the present models implies that some of the relative orientations between the flakes cannot be practically realized. This is consistent with a constricted rotational motion of flakes, indicating a rather congested microenvironment due to the presence of the hyperbranched polymers. The intervention of BPEI molecules between GO also affects the closest distance between the flakes. This can be estimated by means of the radial distribution function (RDF) of GO flakes, considering each flake as a single particle represented by its center of mass. The closest distance can then be determined by the separation corresponding to the first neighbors [71]. Figure 4 shows the calculated RDFs arising from the GO centers of mass in all the examined models.

The discretized form of RDF spectra corroborates the scenario of the spatial confinement of flakes. For systems of both generations, an increase in the polymer content (i.e., going from S3 to S1 and from T3 to T1) results in a shift of the first peak (i.e., the closest separation event between the flakes) to larger distances. Figure S3 shows the dependence of the closest GO separation event, based on the center of mass, on the polymer content. It is noted that the size of the closest GO separation event increases almost linearly with the HBPEI content and that at a fixed polymer wt%, the shortest separation is larger in systems with the smaller polymer. Therefore, it appears that the lower-generation HBPEIs are more effective at creating a larger separation between the GO flakes and their first neighbors (more pronounced in the systems with a higher polymer content). A comparison between the shortest GO separation observed in the S3 and T3 systems with that characterizing aqueous GO dispersion with flakes identical to the ones used here and at a similar water content, i.e., ~10%wt [19], shows that the presence of branched polymers imparts an increase of approximately 70%.

**Figure 5:**
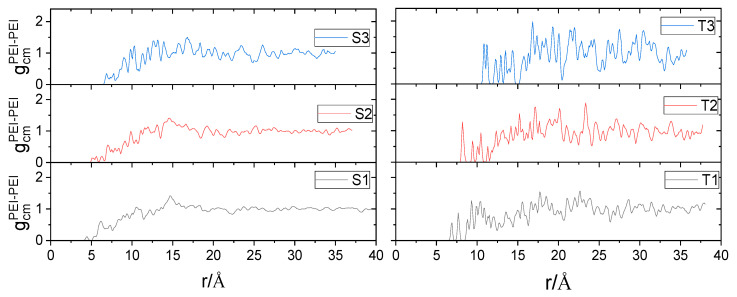
Radial distribution functions arising from the centers of mass of BPEI molecules.

A comparison of RDFs arising from the centers of mass of HBPEI molecules is shown in Figure 5. The length of the shortest separation between the centers of mass of the branched polymers is larger in the third generation systems as compared to that of the second generation models with a similar polymer content (i.e., T1 with S1, T2 with S2 and T3 with S3). This is in line with the larger size of the former one.

In addition, it is shown that in the systems with the larger BPEI, an increase in the GO content results in a notable increase in the length of the shortest separation between the centers of mass of the polymers. The same effect is weaker in the systems comprising smaller BPEIs. This observation might be related to a higher degree of spatial constriction experienced by the larger branched molecules compared to that of their lower-generation analogues when they are confined between GO flakes.

**Figure 6:**
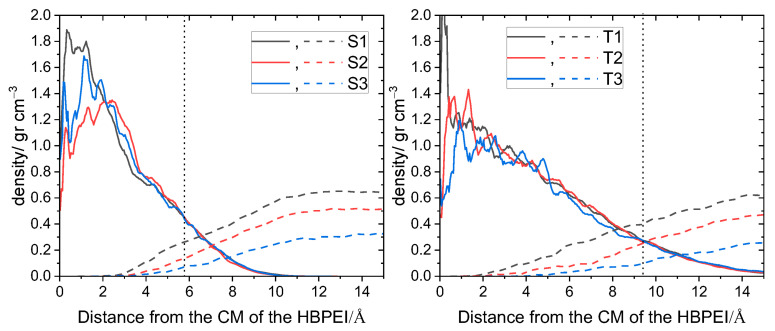
Density profiles of the branched polymers with respect to the center of mass of a BPEI molecule (all BPEI polymers were considered as reference molecules, and averaging was performed). Solid lines represent the density profile of an individual molecule and dashed lines the density arising from all the other polymers. The vertical dotted line approximately denotes the average radius of gyration of a BPEI polymer.

It is also noteworthy that the lowest degree of separation between the centers of mass of BPEI molecules in the systems with the higher content of polymer (i.e., S1 and S2; T1 and T2) lies below the corresponding radius of gyration (see Table S1 in the Supplementary Material). This implies a certain degree of interpenetration between neighboring polymers. Figure 6 illustrates the overlapping of density profiles between neighboring BPEI molecules. The profiles have been constructed by taking each individual polymer as a reference and calculating both its own density and the density of the other polymers as a function of the distance from the center of mass of the reference molecule. The degree of interpenetration is shown to increase upon the increase in polymer content and of the size of the branched molecules. The higher degree of interpenetration that is observed in the larger BPEIs is consistent with a higher degree of confinement experienced by these polymers.

**Figure 7:**
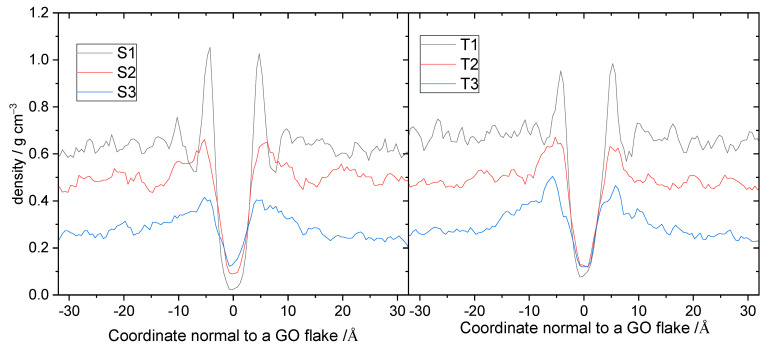
Average density profiles of the BPEI molecules in a direction normal to a GO flake. Coordinate 0 denotes the location of a GO plane.

Furthermore, to examine the spatial arrangement of branched molecules close to the GO surface, we calculated the average density profiles of polymers along a direction normal to a GO flake, as shown in Figure 7. In the systems of both generations, the density increased upon increasing the polymer content, while a peak developed close to the GO surface. At comparable polymer weight fractions, the profiles of the polymers belonging to the two different generations are very similar (see also Figure S4).

The similarity indicates that the packing of polymers of the second and the third generation near the GO surface is practically independent of the size of the polymers. This can be attributed to the deformable structure of the low-generation polymers examined here, which enables their efficient packing close to the GO surface. The presence of a peak close to the GO surface shows a preference of the polymer to be located close to a flake, implying favorable interactions between the two components. This notion is corroborated by the fact that the intensity of the peak grows well above the average density level upon increase in the polymer content.

### 3.2. Hydrogen Bonding

Apart from the electrostatic interactions between oppositely charged components, the fact that both the BPEI molecules and the GO flakes bear hydrogen-bonding donors and acceptors allows the formation of hydrogen bonds, which enhances the stability of the composite’s structure [42]. Actually, hydrogen boding has been identified as the main driving force responsible for increasing the polymer loading in these composites [36]. To elaborate more on this issue, we have estimated the degree of hydrogen bonding between the different components and quantified the effects of the composition and the size of the polymer on hydrogen bond formation.

**Figure 8:**
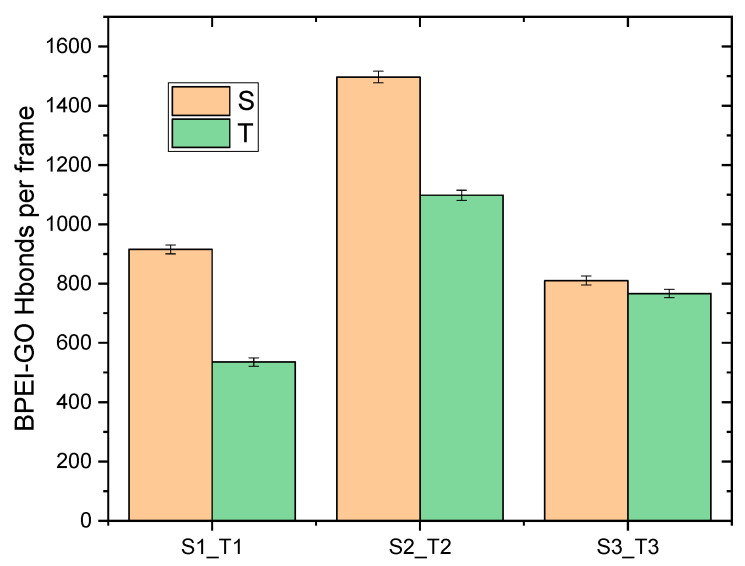
The average number of hydrogen bonds between the BPEI molecules and the GO flakes per frame for the systems based on 2nd (S) and 3rd (T) generations.

Hydrogen bonding was detected using the appropriate analysis tool from the VMD program [72]. The definition of a hydrogen bond was based on distance and angle geometric criteria [73]. Namely, that the donor–acceptor distance did not exceed 3.5 Å and the donor–hydrogen–acceptor angle was larger than 150°. Figure 8 presents the number of hydrogen bonds per frame formed between the GO flakes and BPEI polymers.

Comparing the number of hydrogen bonds in systems with similar polymer weight fraction, but based on different generations (i.e., S1 with T1, S2 with T2 and S3 with T3), shows that in the systems with a high polymer content, those based on the lower generation BPEIs form a larger number of hydrogen bonds with GO. The main contribution in hydrogen bonding between the two components arises from the donors belonging to branched molecules (see Figure S5), and from those, the majority involve nitrogen atoms of protonated primary amines. Therefore, the difference observed between the systems of two generations at similar overall polymer contents could be related to the ability of the lower-molecular-weight polymers to assume more appropriate conformations near the GO surface in order to fulfil the geometric criteria required for hydrogen bonding with the oxidized groups of GO.

Another common attribute in systems of both generations is that the degree of hydrogen bonding between the polymers and the flakes appears to depend in a non-monotonic manner with the GO:BPEI proportion. Here, it appears to be higher in the systems where the GO:BPEI proportion is closer to 1:1 (it should also be mentioned that in these systems, the water percentage is lower compared to that of the rest of them). An estimation of the non-bonded energy between the GO flakes and the polymers indicates more favorable interactions at this composition, as presented in Table 2.

As shown, irrespective of polymer generation, both the van der Waals and the electrostatic interactions appear to be more favorable in the S2/T2 systems. This observation implies that there can be a mixing proportion at which the interfacial interaction between the two components is optimized. The decrease in the degree of hydrogen bonding upon the further increase in GO content could be associated with the departure from that optimal composition.

**Figure 9:**
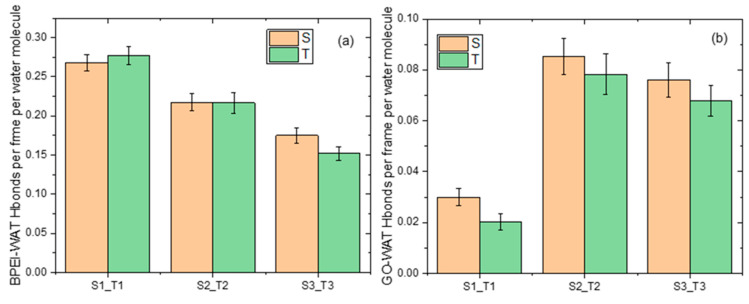
The number of hydrogen bonds per frame and per water molecule between water and (**a**) the polymer and (**b**) the GO flakes in the examined systems.

Apart from the role of hydrogen bonding in polymer/GO association, it is interesting to examine its role in the water-mediated hydrogen-bonding network formed within the composites. The latter one contributes also to the structural stability of these materials [60]. Figure 9 displays the degree of hydrogen bonding between the polymer and the GO flakes with water.

**Figure 10:**
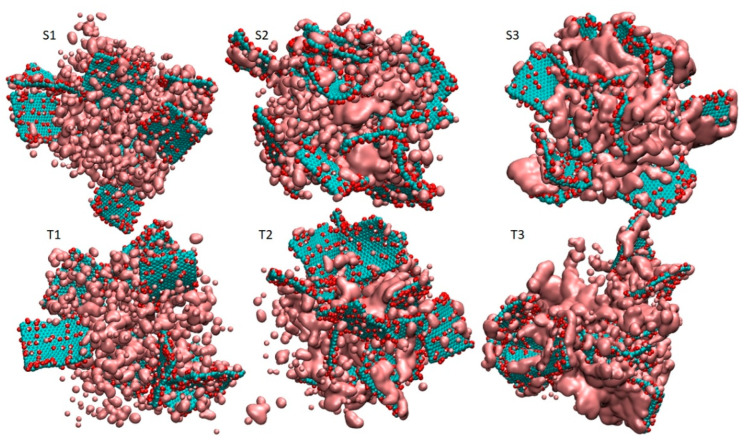
Snapshots of the water accessible surface areas (see text). All the other moieties, except the GO flakes, are omitted for clarity. Carbon atoms of the GO flakes appear in dark cyan, oxygen atoms in red and hydrogen atoms in white. The surface accessible area appears in dark pink.

The degree of polymer–water hydrogen bonding expectedly decreases as the polymer content is lowered. At a high polymer content (i.e., in systems S1 and T1), the degree of hydrogen bonding between GO and water is very small. This should be related to the fact that at the same composition, the availability of hydrogen-bonding-capable sites of the polymer that can form hydrogen bonds with water is at its maximum. Therefore, the low degree of GO/water hydrogen bonding in these systems could be associated with the antagonistic action of the polymer in forming hydrogen bonds with water. The significant increase in the degree of GO/water hydrogen bonding observed in systems S2 and T2 can be justified if we take into account that the lowering of the polymer content reduces the number of hydrogen-bonding-capable sites between the BPEIs and water, and thus, increases the availability of water molecules to form hydrogen bonds with GO. In addition, those water molecules already associated with BPEIs that are located close to a GO surface possess a higher probability of staying longer at a position close to the same GO surface, and thus, they form hydrogen bonds with the flake. At an even lower polymer content (i.e., systems S3 and T3), although a number of water molecules were kept close to the GO surface due to hydrogen bonding with the adsorbed polymers drops, the fraction of water molecules available to form hydrogen bonds with GO increases, as does the GO surface accessible to water molecules, resulting also in high levels of GO/water hydrogen bonding. Figure 10 portrays snapshots of the surfaces accessible to water molecules in the examined models.

The calculation of accessible surface area to a probe molecule was performed using a water radius of 0.14 nm [74] according to the double cubic lattice method [75], as implemented in GROMACS [61]. At the higher polymer content (systems S1 and T1), the surface accessible to water molecules is distributed in small islands mostly away from the GO flakes. As the polymer content drops (systems S2 and T2) larger patches of water, accessible surfaces appear adjacent to the GO flakes, while at the lower-polymer-content models, S3 and T3, the accessibility of the GO surface increases significantly, as indicated by the larger accessible areas covering the flakes. The quantification of the trajectory-averaged water accessible area (see Figure S6) shows that this increases in a monotonic manner for systems of both generations as the polymer content drops.

### 3.3. Water and Ion Transport

**Figure 11:**
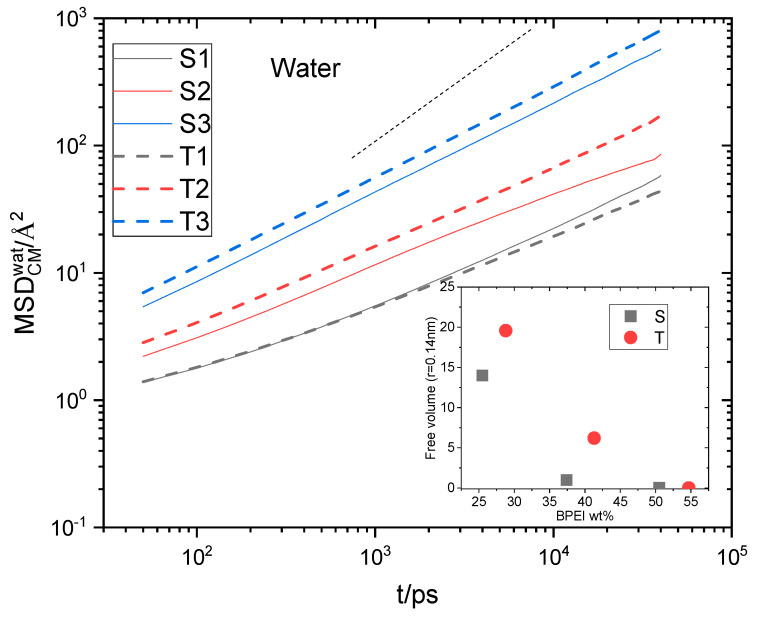
Main panel: MSD arising from the centers of mass of the water molecules. The dotted short line denotes a slope of 1. Inset: free volume accessible to a probe with a radius of 0.14 nm in the examined systems as a function of the polymer content. Error margins are smaller than the size of the symbols.

Water and ion transport is of special interest in composite materials similar to those studied here since they are related directly to their performance in relevant applications [76,77,78]. In particular, for graphene oxide dispersions at low-level humidity conditions where hydrogen bonds are expected to be weaker [79], the diffusional motion of water molecules could play a significant role in the formation or disruption of the hydrogen-bonding network. To check the effect of polymer generation and of the composition of the examined systems in water transport, we have calculated the Mean Squared Displacement (MSD) of the centers of mass of water molecules, as shown in Figure 11. Water transport in all systems is subdiffusive (compare the late-time slopes of the curves with the slope of unity). This is to be expected since water diffuses within a highly heterogeneous environment and under conditions of spatial confinement [76,79,80,81]. For systems of both generations, the MSD increases as the polymer content decreases. In addition, it appears that in the lower-polymer-content systems, water diffuses slightly faster in those with the larger HBPEIs. The increase in water transport upon the decrease in the polymer content is consistent with the fact that the number of hydrogen bonds between water and polymer decreases (see Figure 9a). Moreover, the calculation of the average free volume of the systems [82] presented in the inset of Figure 11 shows that this increases when the content in the polymer drops. At the same time, however, as discussed earlier, the number of hydrogen bonds between water and GO increases, which is expected to significantly slow down the diffusion of water molecules [76]. To resolve this issue, we performed the calculation of the self Van Hove functions [71] originating from the centers of mass of water.

**Figure 12:**
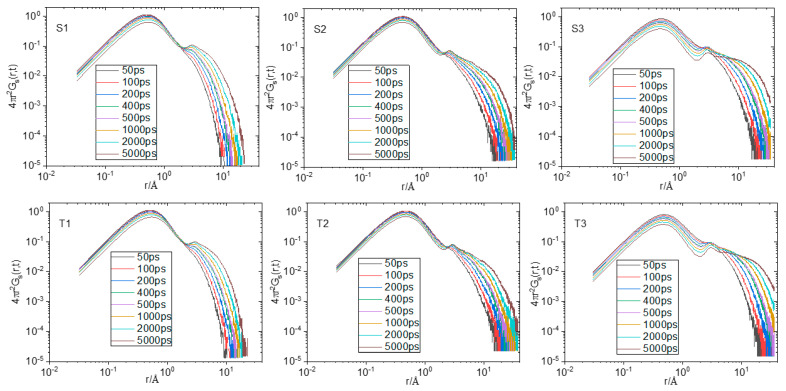
Self Van Hove functions arising from the center of mass of the water molecules at different time periods.

The time–space self van Hove function maps the distribution of distances travelled by the particles under examination (here, the centers of mass of water molecules) within a specific time period. This enables the distinction of particle populations possessing different mobilities. The self Van Hove function is given by the expression:

(1) $$G{}_{s}\left(r,t\right)=\frac{1}{N}\langle \sum_{i}\delta \left[r-\left|r{}_{i}(t)-r{}_{i}(0)\left.\right|\right.\right.\left.\right]\rangle$$

where *r_i_*(0) and *r_i_*(*t*) represent the position vector of particle *i* at times 0 and *t*, respectively, *r* corresponds to a specific distance, *N* is the total number of particles, the angle brackets denote time and ensemble average and *δ* is the Dirac function. The self Van Hove function is proportional to the probability that a particle is at position r at time t, given that the same particle was at the origin (*r* = 0) at time *t* = 0. Figure 12 shows the self Van Hove functions of the centers of mass of water molecules at different time spans.

The common features characterizing all systems are the presence of a main peak at a short distance whose location remains insensitive throughout time, a broad peak appearing as “shoulder”, which shifts to larger distances at longer timescales, and an indication of a minimally intense peak at intermediate distances (more prominent in the systems with the lower polymer content). The short-distance peak is consistent with a water population whose molecules remain constricted, travelling thus only a very small distance over time. The broad peak shifting to longer distances over time indicates a water population with much more freedom to diffuse. The group of waters contributing to the short-distance peak can thus be identified as a “bound” population [76], which is consistent with those molecules forming hydrogen bonds with a much slower-moving component, such as the polymer, the GO flakes, and possibly, the hydrated ions. The broad peak should therefore be associated with “unbound” water molecules, which can diffuse much faster. Although the intensity of the peak describing the “bound” group is higher, implying that the number of constricted waters is larger compared to the “unbound” group, a weighted average of the distance travelled by all water molecules can yield an increase in the MSD due to the much larger distance travelled by the “unbound’ population. In other words, the main cause of the increase in the average MSD of water should arise from those molecules belonging to the “unbound” population. If we compare the spectra at a constant timescale (see Figure S7), it appears that the broad peak shifts to longer distances as the polymer content drops. Since the broad peak shifts to longer distances upon decrease in the polymer content, the average MSD should increase, which would be in line with the behavior noted in Figure 11. At a similar polymer content, the somewhat larger MSD of the water observed in the systems with the larger polymer is consistent with the higher percentage of water-accessible free volume in these systems (see the inset in Figure 11).

**Figure 13:**
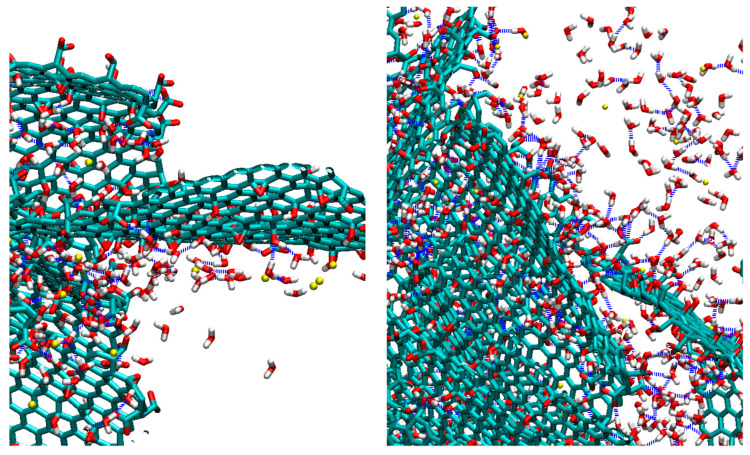
Snapshots of the S3 (**left**) and the T3 (**right**) systems. Carbon atoms are shown in dark cyan, oxygen atoms are shown in red, hydrogen atoms are shown in white and Chlorine atoms are shown in yellow (note that the atoms are not represented by their VDW radii). The blue stacked short lines indicate hydrogen bonds.

Regarding the origin of the intermediate peak, which indicates the presence of a “loosely bound” water population, a plausible assumption is that it could originate from water molecules, which form hydrogen bonds with “bound” waters. The separation between the locations of the “bound” and the “loosely bound” peaks (i.e., between 2.5 Å and 3 Å) is commensurate to a distance of 2.8 Å, which corresponds to the closest neighbors in the water–water rdf function (see Figure S8). Figure 13 shows snapshots of the S3 and T3 systems, where these hydrogen bonds are visible.

**Figure 14:**
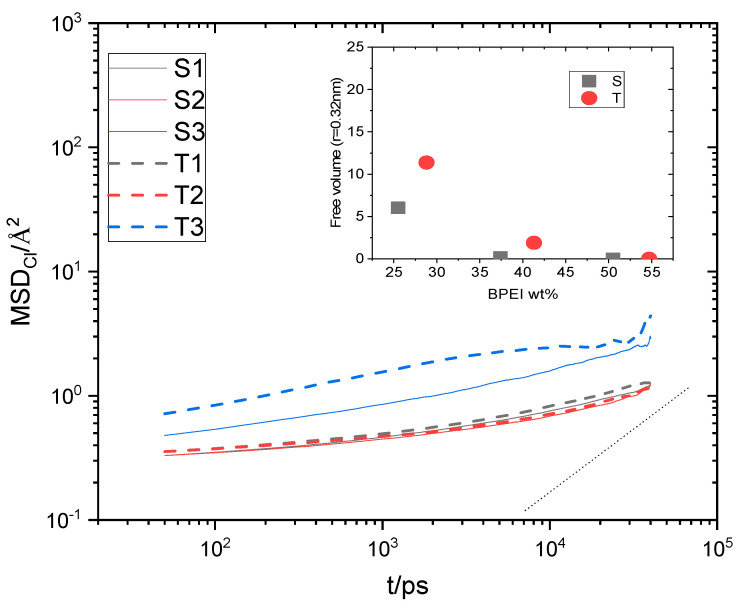
The MSD of the Chlorine ions in the examined systems. The inset shows the average free volume accessible to a probe particle with radius of 3.2 Å, corresponding to the hydration radius of Chlorine. The error margins are within the size of the symbols. The short, dotted line represents a slope of 1.

To monitor the diffusional motion of the Chlorine counterions, we studied their MSD behavior, as shown in Figure 14.

As was the case for water, the transport of Chlorine ions in the constricted environment of the composites is subdiffusive. Ionic transport appears to be much slower compared to that of water molecules (see Figure 11) for all the examined systems. For systems of both BPEI generation types, ionic motion is remarkably slow for the two higher-polymer-content composites (i.e., S1 and S2; T1 and T2), while a sudden increase in Chlorine MSD is observed in the lower-polymer-content models (i.e., S3 and T3). This behavior can be accounted for if we consider that Chlorines essentially diffuse as hydrated ions. The free volume accessible to the hydrated ions can be estimated if we use a particle with radius of 0.32 nm as a probe. This value corresponds to the first peak of the water–Chlorine rdf (see Figure S9) and is known to represent the hydration radius of the Chlorine ions [83]. The result of this estimation is shown in the inset in Figure 14. Apparently, the free volume for the hydrated ions is very low in the systems with a higher polymer content, but it increases significantly in systems S3 and T3. This behavior is consistent with the very low MSD values observed in systems S1 and S2 and T1 and T2 and its stepwise increase in systems S3 and T3. The difference noted in Chlorine MSDs between the S3 and the T3 models is also consistent with the difference in the percentage of free volume experienced by the hydrated ions in these composites. The higher percentage of free volume in the systems with the larger polymers could be associated with the different packing of molecules within the 3D structure of the composite at high GO contents. This is also reflected in the somewhat lower density of these systems compared to that of the composites with a higher polymer content (see Figure S10).

## 4. Conclusions

In this work, we have examined, in detail, nanocomposites comprising graphene oxide and two different generations of hyperbranched poly(ethyleneimine) at low hydration and at neutral pH conditions. We monitored the effects of the size of the branched polymers and of the composition on the morphological characteristics of the composite structure, on the affinity between the GO flakes and the polymeric molecules and on water and ion dynamics.

It was found that the presence of the branched polymers hindered the formation of stacked GO configurations. The separation between the centers of mass of GO flakes was found to depend on the examined composition in systems of both polymer sizes, but the lower-molecular-weight polymers were found to be more effective at modifying interflake separation due to their more efficient packing in the composite on account of their smaller size.

The examination of interfacial interactions between the polymer molecules and GO provided indications about the presence of an optimal mixing composition in which the energetic affinity between the composites is maximized. The strong affinity between the main components of composites is expected to lead to materials with increased structural stability. In our case, this composition was found to be at almost 1:1 in wt% mixing proportions between the polymer and the GO flakes and with a water content between 5wt% (T2) and 6.7wt% (S2). The polymeric molecules were found to form a much higher number of hydrogen bonds with water compared to those formed between water the GO flakes due to the much higher number of donors they could dispose of. Upon the decrease in the polymer content, the water–GO hydrogen bonds increased markedly because of the increase in the size of the water accessible area close to the GO flakes and the higher availability of water molecules to form hydrogen bonds. The water-mediated hydrogen-bonding network between the components of the mixture is expected to play a significant role in the enhancement of the mechanical properties of these composites [33]. Τhe water percentage corresponding to the systems with the higher GO/BPEI affinity (i.e., S2 and T2) is close to that which was found to lead to an enhanced stiffness of graphene oxide/poly(vinyl) alcohol nanocomposite papers [33].

Water transport within the formed structure was found to largely depend on the composition for the systems of both generations, but as the polymer content decreased, a larger increase in the average rate of water diffusion was observed in the composites with the larger polymer. This was found to be correlated with the higher percentage of free volume available in these systems due to their less-effective packing. A detailed analysis of the self-diffusion modes of water molecules revealed the presence of water populations with distinctly different mobilities: a “bound” population formed by water molecules hydrogen bonded directly to the less-mobile components of the composite, a “loosely bound” population consistent with water molecules hydrogen-bonded with the “bound” waters, and an “unbound” population with much more mobility. The dependence of the average rate of water transport on composition was essentially caused by the behavior of the “unbound” population.

Ionic transport was found to strongly depend on the composition of systems of both generations. A higher polymer content resulted in a very limited level of ionic diffusivity due to the very low percentage of free volume available to hydrated ions. A stepwise increase in ionic motion was observed when the polymer content dropped to the lower value examined (here, approximately lower than 30%wt), indicating that in these systems, there is a polymer concentration threshold above which ionic transport is strongly hindered. As was the case in water, higher ionic diffusivity was observed in the system with the larger polymers, owing to their less-efficient packing, which resulted in a higher percentage of available free volume for hydrated ions. This information could be of particular interest in applications where the control of ionic conductivity is desired.

Overall, we believe that the detailed information provided in this work regarding the role of polymer size and composition in controlling parameters, such as flake separation, energetic stability, and the degrees of water and ionic transport, will provide a valuable insight into the potential use of these materials in a wide range of applications, including nanofiltration [23,45,46,49,84], sensing [52,85], gas separation [86,87], and controllable ion transport processes [88,89].

## Figures and Tables

**Table 1:** Composition of the simulated models. The models denoted as S1, S2 and S3 are based on the 2nd generation polymer (HBPEI600) and those denoted as T1, T2 and T3 models on the 3rd generation polymer (HBPEI1800) (see Figure 1).

System	Number of BPEI Molecules	Number of GO Flakes	Number of Water Molecules	Number of Cl^−^ Counterions	wt% in PEI	wt% in GO	Total Number of Atoms	Average Box Size (nm)
S1	300	10	1877	1840	50	23	51,391	7.9
S2	198	18	1240	918	37	46	42,550	7.5
S3	100	18	1241	232	25	62	28,891	7.0
T1	100	10	1500	1140	55	25	48,160	7.6
T2	72	18	900	540	41	48	41,436	7.6
T3	38	17	1385	90	29	60	29,669	7.2

**Table 2:** Non-bonded energy contributions between the GO flakes and the BPEI polymers (kJ/mol).

2nd Generation			
Systems	S1	S2	S3
vdW (kJ/mol)	−13,699.3 ± 222.7	−15,546.5 ± 264.2	−8273.7 ± 252.9
Coulomb (kJ/mol)	−109,939.4 ± 436.0	−186,883.2 ± 501.0	−163,141.1 ± 509.0
Total (kJ/mol)	−123,638.7 ± 658.7	−202,429.7 ± 765.2	−171,414.8 ± 761.9
3rd Generation			
Systems	T1	T2	T3
vdW (kJ/mol)	−16,314.0 ± 215.8	−19,814.6 ± 289.9	−12,409.8 ± 245.1
Coulomb (kJ/mol)	−104,374.0 ± 403.1	−179,628 ± 502.6	−143,286.0 ± 500.7
Total (kJ/mol)	−120,688 ± 618.9	−199,442.6 ± 792.5	−155,695.8 ± 745.8

## Data Availability

The data can be shared up on request.

## Supplementary Materials

The following supporting information can be downloaded at: https://www.mdpi.com/article/10.3390/nano13121865/s1, Figure S1: Initial configurations of the simulated systems. GO flakes appear in dark cyan, HBPEI molecules in green, oxygen atoms in red, hydrogen atoms in white and Cl^−^ counterions in yellow; Figure S2: (a,b) Radius of gyration (Rg) of polymer chains as a function of time for S1,S2,S3 and T1,T2,T3 respectively; (c,d) Average pair distance between the center of mass of GO flakes as a function of time for S1,S2,S3 and T1,T2,T3 respectively; Figure S3: The shortest separation between the centers of mass of the GO flakes as a function of the systems’ content in polymer; Figure S4: Comparison of the density profiles of the BPEI polymers in systems with comparable polymer content, along a direction normal to a GO flake; Figure S5: Comparison of the contribution from BPEI and GO donors to the total number of the hydrogen bonds formed between them; Figure S6: Solvent access surface area for water molecules in the examined models; Figure S7: Comparison of the self Van Hove functions arising from the centers of mass of water at a constant timescale of 1000ps; Figure S8: The radial distribution function arising from the centers of mass of the water molecules. The main peak is centered at 2.8Å; Figure S9: The radial distribution function arising from the Chlorine ions. The main peak is centered at 3.2Å; Figure S10: Average density of the simulated models; Table S1: Average radius of gyration of the HBPEI polymers in the examined systems.
