# Supplementary material for: Morphology and Dynamics in Hydrated Graphene Oxide/Branched Poly(ethyleneimine) Nanocomposites: An In Silico Investigation
Source: Nanomaterials (Basel). 2023 Jun 15;13(12):1865. doi: 10.3390/nano13121865 (PMC10303114; doi:10.3390/nano13121865)
Supplement: Supplementary file 1 [file nanomaterials-13-01865-s001.zip › nanomaterials-2433265-supplementary.pdf]

Supplementary Material

Morphology and Dynamics in Hydrated Graphene-Oxide/Branched Poly(ethyleneimine) Nanocomposites: an in-Silico Investigation

Anastassia Rissanou [1], Apostolos Konstantinou [2] and Kostas Karatasos [2,*]

1 Theoretical & Physical Chemistry Institute, National Hellenic Research Foundation, 48 Vassileos Constantinou Avenue, 11635 Athens, Greece; trissanou@eie.gr

2 Department of Chemical Engineering, University of Thessaloniki, P.O. BOX 420, 54124 Thessaloniki, Greece

* Correspondence: kkaratas@cheng.auth.gr

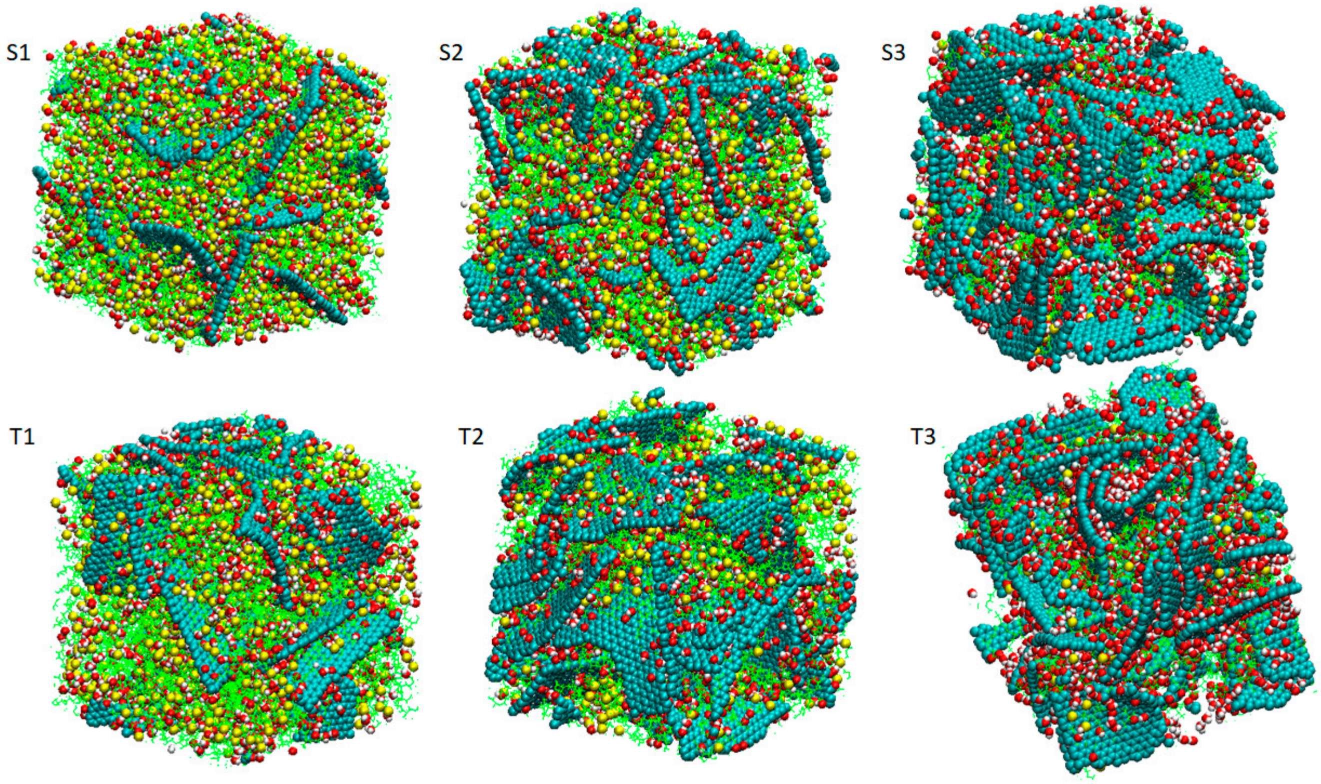

Figure S1. Initial configurations of the simulated systems. GO flakes appear in dark cyan, HBPEI molecules in green, oxygen atoms in red, hydrogen atoms in white and Cl^- counterions in yellow

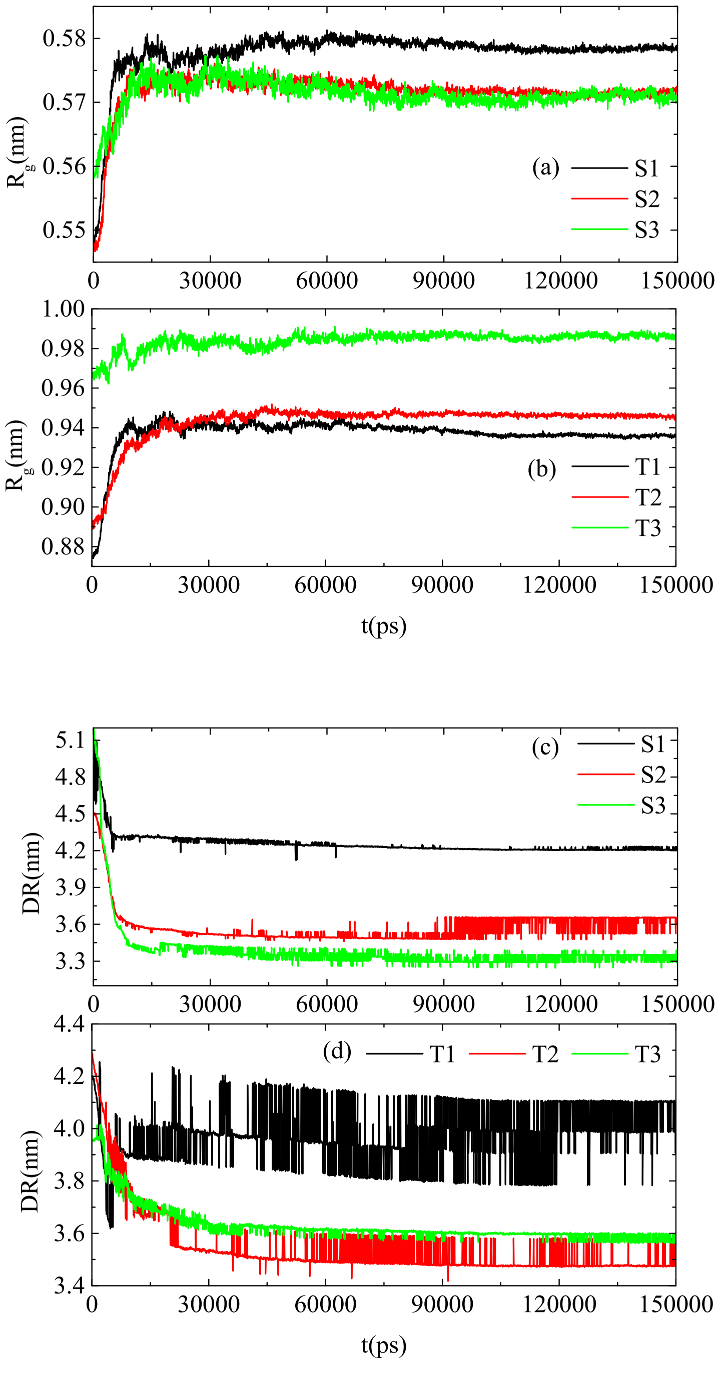

Figure S2. (a, b) Radius of gyration (R_g) of polymer chains as a function of time for S1,S2,S3 and T1,T2,T3 respectively; (c, d) Average pair distance between the center of mass of GO flakes as a function of time for S1,S2,S3 and T1,T2,T3 respectively.

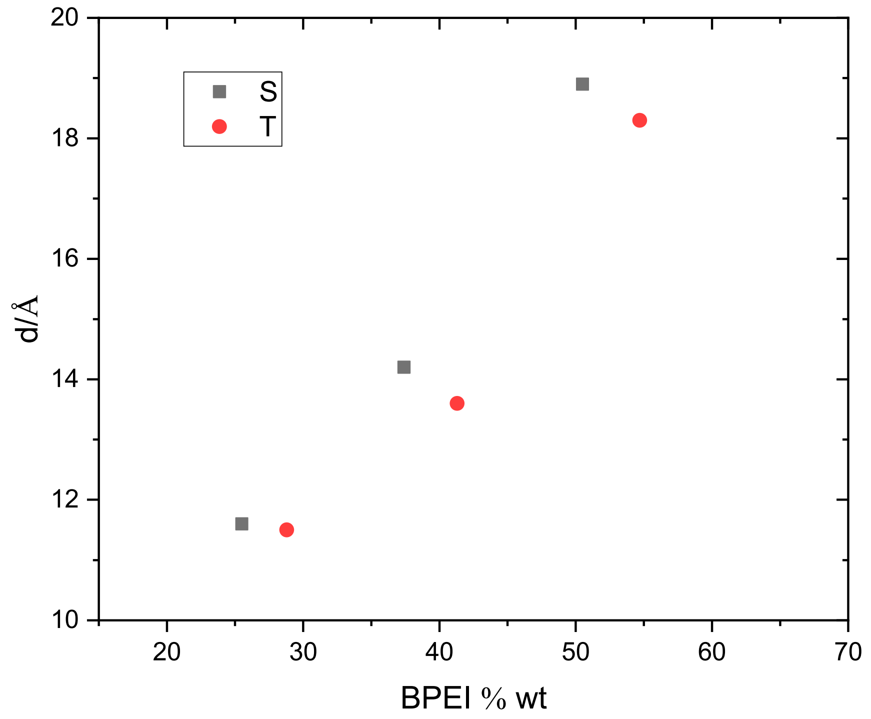

Figure S3. The shortest separation between the centers of mass of the GO flakes as a function of the systems' content in polymer.

Table S1. Average radius of gyration of the HBPEI polymers in the examined systems

System	R_g(Å)
S1	5.775±0,003
S2	5.705±0,004
S3	5.690±0,007
T1	9.332±0,006
T2	9.421±0,006
T3	9.823±0,010

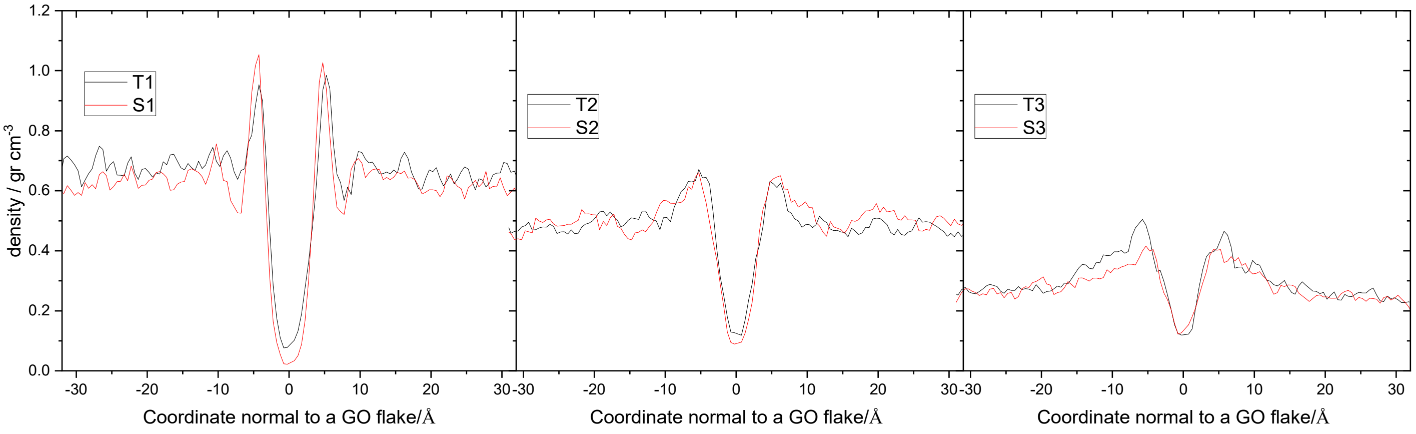

Figure S4. Comparison of the density profiles of the BPEI polymers in systems with comparable polymer content, along a direction normal to a GO flake

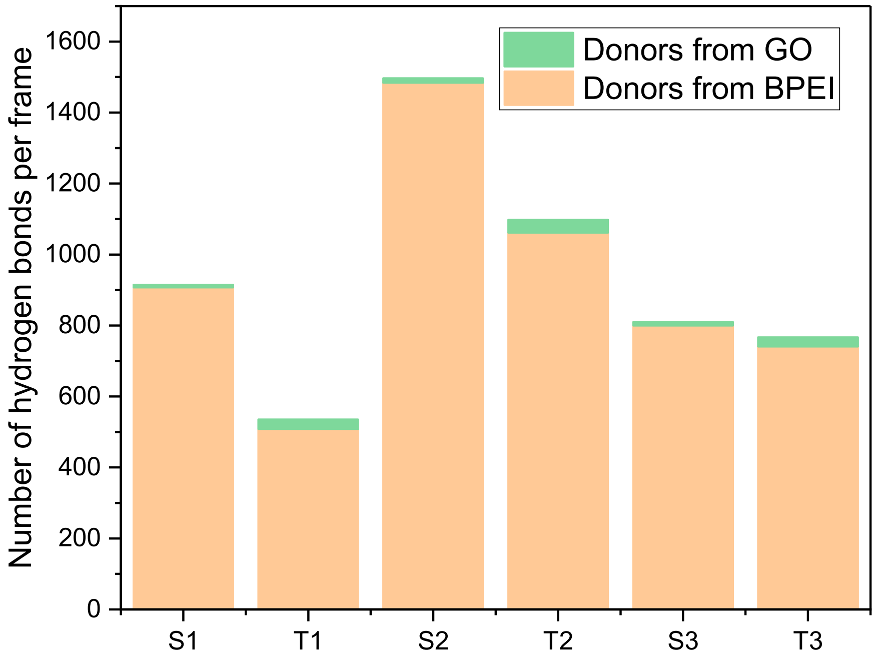

Figure S5. Comparison of the contribution from BPEI and GO donors to the total number of the hydrogen bonds formed between them.

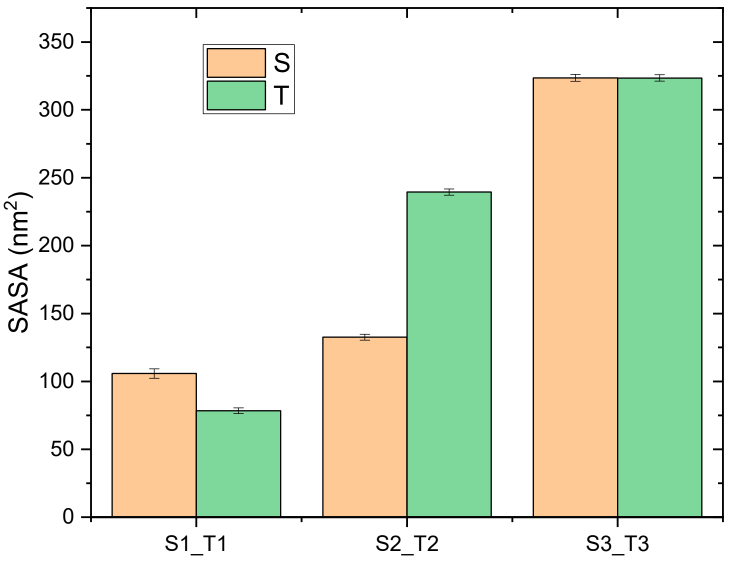

Figure S6. Solvent access surface area for water molecules in the examined models

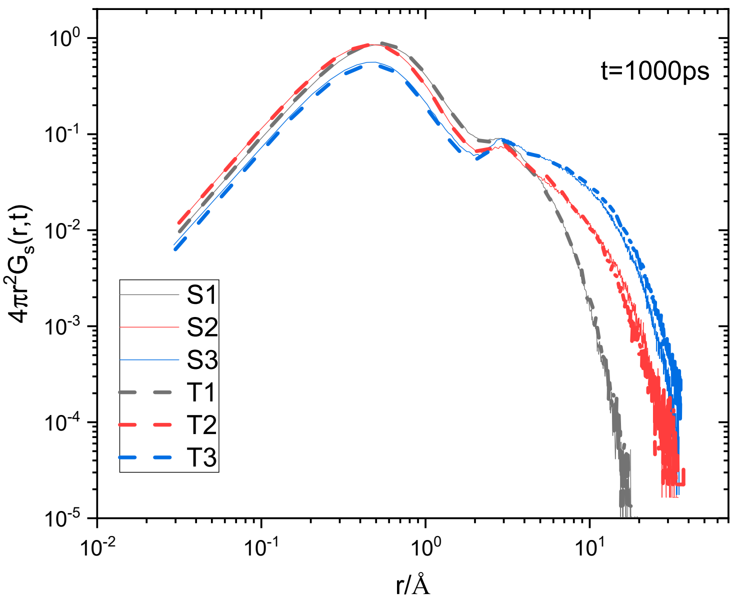

Figure S7. Comparison of the self Van Hove functions arising from the centers of mass of water at a constant timescale of 1000ps

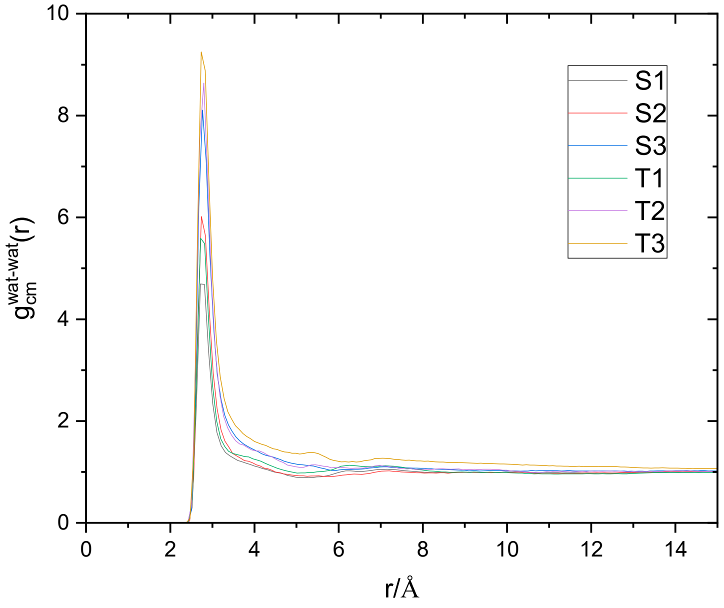

Figure S8. The radial distribution function arising from the centers of mass of the water molecules. The main peak is centered at 2.8Å

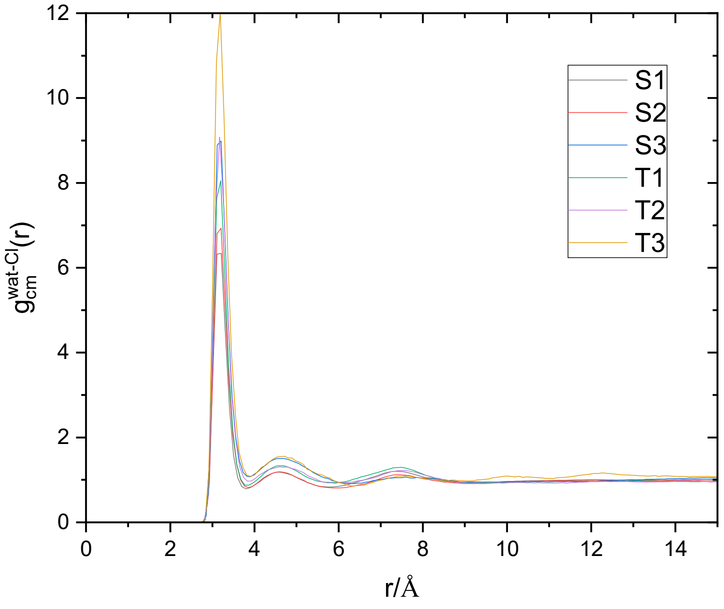

Figure S9. The radial distribution function arising from the Chlorine ions. The main peak is centered at 3.2Å.

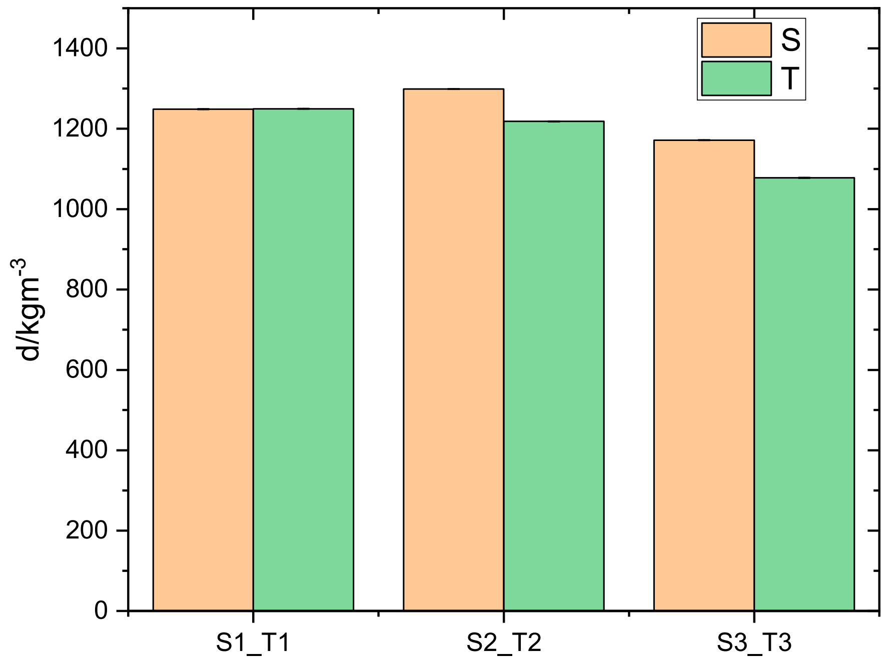

Figure S10. Average density of the simulated models.
